# Antifungal activity of protein hydrolysates from Thai Phatthalung Sangyod rice *(Oryza sativa* L.) seeds

**DOI:** 10.14202/vetworld.2023.1018-1028

**Published:** 2023-05-13

**Authors:** Juthatip Jeenkeawpieam, Prawit Rodjan, Sittiruk Roytrakul, Kritsada Pruksaphon, Watcharapong Mitsuwan, Noppharat Tanthanathipchai, Chaiwat Boonkaewwan, Irma Tedja, Monsicha Pongpom

**Affiliations:** 1 Akkhraratchakumari Veterinary College, Walailak University, Nakhon Si Thammarat 80160, Thailand; 2 One Health Research Center, Walailak University, Nakhon Si Thammarat, 80160, Thailand; 3 School of Agricultural Technology and Food Industry, Walailak University, Nakhon Si Thammarat, 80160, Thailand; 4 Functional Ingredients and Food Innovation Research Group, National Center for Genetic Engineering and Biotechnology, Pathum Thani 12120, Thailand; 5 Department of Microbiology, Faculty of Medicine, Chiang Mai University, Chiang Mai 50200, Thailand; 6 Department of Biochemistry and Molecular Biology, Monash Biomedicine Discovery Institute, Monash University, Victoria 3800, Australia

**Keywords:** antifungal peptide, *Oryza sativa* L, Phatthalung Sangyod rice, protein hydrolysate, Thai rice seed, zoonoses

## Abstract

**Background and Aim:**

Fungal zoonoses are an economic and public health concern because they can cause various degrees of morbidity and mortality in animals and humans. To combat this issue, alternative natural antifungals, such as products derived from rice protein hydrolysates or rice antifungal protein/peptide are being considered because they are highly bioactive and exhibit various functional properties. Thailand is a leading rice producer and exporter. Among the various cultivated rice varieties, Sangyod rice (*Oryza sativa* L.) is exclusively indigenous to Thailand’s Phatthalung province; it has a Thai geographical indication tag. Here, we investigated whether the Phatthalung Sangyod rice seeds have bioactive antifungal peptides.

**Materials and Methods:**

Antifungal activity in four Sangyod rice seed extracts (SYPs) – namely, (1) the crude lysate, SYP1; (2) the heat-treated lysate, SYP2; (3) the heat- and pepsin digested lysate, SYP3; and (4) the heat- and proteinase K-digested lysate, SYP4 – was analyzed. Protein concentrations in these SYPs were determined using the Bradford assay. The total phenolic compound content was determined using the modified Folin–Ciocalteu method in a 96-well microplate. Then, the SYP protein pattern was determined using the sodium dodecyl sulfate-polyacrylamide gel electrophoresis. Subsequently, using the agar well diffusion method, the antifungal properties of these SYPs were tested against ten medically important pathogenic fungi. The minimal inhibitory concentration (MIC) and minimal fungicidal concentration values were determined for the active SYPs – SYP2-4. Finally, the clinical safety of SYP4 was determined using a hemolytic assay (using canine red blood cells [RBCs]).

**Results:**

The crude lysate SYP1 did not show antifungal activity against any of the ten tested pathogenic fungi. Surprisingly, hydrolysates SYP2, SYP3, and SYP4 displayed antifungal properties against the ten tested pathogenic fungi. Thus, heat and enzymatic hydrolysis seem to transform the bioactivity of the crude protein extract – SYP1. Further, SYP4 shows the most effective antifungal activity. It completely inhibited *Cryptococcus neoforman*s, *Talaromyces marneffei* yeast phase, *Trichophyton mentagrophyte*s, and *Trichophyton rubru*m. A partial inhibitory action on *Candida albican*s and *Microsporum gypseum* was possessed while showing the least activity to *C. neoforman*s. SYP4 was nontoxic to canine RBCs. Hemolysis of canine RBCs was undetectable at 1 × MIC and 2 × MIC concentrations; therefore, it can be safely used in further applications.

**Conclusion:**

These results indicate that heat and proteinase K hydrolyzed SYP is a very potent antifungal preparation against animal and human fungal pathogens and it can be used in future pharmaceuticals and functional foods.

## Introduction

Rice, one of Thailand’s most important economic plants, is a rich source of protein and other nutrients [1, 2]. Rice protein has distinct nutritional and hypoallergenic properties compared to cereal and legume proteins. Sangyod red rice (*Oryza sativa*, L., var. indica) is a unique red rice variety grown for over a century in Phatthalung, a southern province. It has been protected under the law and registered as a geographical product since 2006 [3]. Sangyod has small, elongated grains, and brown variety rice has the highest nutritional value [4]. The cooked rice is tender and fragrant. Minerals, vitamin B complex, and bioactive compounds are abundant [5]. In addition, the antioxidant activity of the water extract of Sangyod red rice is 3× higher than that of the jasmine white rice [6]. Consistently, it is high in phenolic compound concentrations and flavonoid activities [7]. Natural antioxidants protect against several oxidative stress-related chronic diseases, such as diabetes, cardiovascular disease, and cancer [8]. Recently, the focus has shifted to the phytochemical properties of Sangyod rice seed and bran. Being rich in vitamins, phytochemicals, and antioxidants, Sangyod rice is beneficial to human health [6, 7].

The growing threat of fungal infections is partly attributable to the increase in immunocompromised patients caused by recent medical advances [9, 10]. Moreover, several emerging and re-emerging fungal pathogens, such as Mucorales, *Candida auris*, and certain molds, are difficult to treat as they are intrinsically resistant to current antifungal agents. A previous review highlighted the recent developments in novel antifungal agents and other alternate approaches for fighting against fungal infections. [11–13]. Unfortunately, only three classes of drugs are available for systemic antifungal therapy: polyenes (e.g., amphotericin B), triazoles (e.g., fluconazole), and echinocandins (e.g., caspofungin). Few additional drugs, such as 5-flucytosine, are available as adjunctive treatments. Therefore, scientists are investigating bioactive peptides to serve as potential replacements [14, 15]. Biologically active peptides are small pieces of large precursor proteins that are inactive until being fragmented apart. When proteolytic enzymes break them apart, they can interact with the right receptors and regulate physiological functions [14, 15]. They exhibit antihypertensive, anticoagulant, antithrombotic, antiproliferative, antioxidant, antifungal, antibacterial, antiviral, and immunomodulating properties [14, 15]. This study is interested in discovering novel bioactive peptides in the protein hydrolysates of Thai rice seeds. Protein hydrolysate – which may contain chemically or enzymatically hydrolyzed active peptides that are frequently attached to natural proteins [15] is one of the sources of antimicrobial peptides or proteins. Antifungal peptides (AFPs) play a major role in the habitation of fungi [16]. Antifungal peptides act as an innate immune response of the host because these peptides can inhibit a wide variety of infectious microorganisms without posing a significant risk to the host [17]. Numerous researchers confirm that plant hydrolysates can release bioactive peptides and are effective in treating various diseases [18–20].

A study revealed the antibacterial activity of Sangyod rice seed [21]. They showed that the pepsin-hydrolyzed protein (extracted from Sangyod rice seed) inhibits the growth of human pathogenic bacteria *Staphylococcus aureus*, *Pseudomonas aeruginosa*, and *Escherichia coli*. Suesattayawong *et al*. [22] reported that Sangyod rice seed is resistant to the *Sarocladium oryzae* fungal infection. However, antifungal property has not yet been reported for Sangyod rice seed extracts or its protein hydrolysates.

Thus, we aimed to study whether protein hydrolysates from Sangyod rice seed, which possess antifungal activity against opportunistic fungal pathogens which cause zoonotic diseases.

## Materials and Methods

### Ethical approval

All research procedures followed recommendations from the Walailak University Institutional Animal Care and Use Committee (WU-IACUC), Walailak University, Thailand (number: WU-ACUC-65037).

### Study period and location

The study was conducted from January 2022 to August 2022. Samples of Sangyod rice seed were purchased from an organic farm located in Phatthalung Province, southern Thailand. Sangyod protein isolation, protein hydrolysate preparation, protein concentration and total phenolic compound content determinations, antifungal activity testing, minimal inhibitory concentration (MIC) and minimal fungicidal concentration (MFC) determination, and the safety of protein hydrolysates were investigated at Walailak University, Nakhon Si Thammarat, Thailand.

### Preparation and isolation of protein hydrolysate from Sangyod rice seed

Fifty grams of Sangyod rice seed were finely ground, homogenized in 10 mM sodium acetate (CH_3_COONa) buffer (pH = 5.2) containing 0.5% polyphenol pyrrolidine (PVP), and centrifuged to remove the phenolic-PVP compound-complex. The supernatant or crude protein extract (SYP1) was collected and the protein and phenolic compound concentrations were determined [23]. SYP1 was treated differently to obtain: (1) SYP1 – The crude untreated extract; (2) SYP2 – SYP1 boiled at 100°C for 10 min; (3) SYP3 – SYP1 hydrolyzed using pepsin at pH = 3.7; and (4) SYP4 – SYP1 hydrolyzed with proteinase K (SYP1 was freeze-dried, dissolved in 1 × phosphate-buffered saline (PBS) [pH = 7.4], and hydrolyzed with proteinase K).

### Determination of protein concentration

The protein concentration was determined using Bradford assay (in a 96-well microplate); bovine serum albumin (BSA) was used as a standard. Bradford assay is based on the shift in absorbance maximum of Coomassie Brilliant Blue G-250 dye from 465 to 595 nm following binding to proteins in solution. Five microliters of SYP1–4 were pipetted into test wells (triplicate for each SYP sample). For the calibration curve, serial solutions of BSA with final concentrations of 0, 2, 4, 6, 8, and 10 μg in 5 μL distilled water were prepared; 5 μL of distilled water was used as a reagent blank. 200 μL of Bradford protein reagent (Bio-Rad Protein Assay; dye reagent concentrate, Bio-Rad, Hercules, CA, USA) was added to each well containing the BSA solutions (of different concentrations) and incubated for 10 min. Then, the absorbance at 595 nm was measured using a microplate reader (BioTek™ Synergy™ H4 Hybrid Microplate Reader, Winooski, VT, USA). The absolute absorbance of the BSA standard (OD_BSA_−OD_blank_) was used to generate a standard graph. The protein concentration in each SYP1–4 sample was determined by comparing the value of OD_sample_−OD_blank_ to the standard graph [24].

### Determination of phenolic compound content

Measurement of phenolic compound content and proteolytic digestion assays was performed to confirm that the antifungal effect was derived from proteins in the SYP1–4 samples. Total phenolic content was determined using the modified Folin–Ciocalteu method (in a 96-well microplate using gallic acid as a standard). Briefly, the 40 μL samples were mixed with 60 μL of 10% Folin–Ciocalteu reagent in the wells of the 96-well microplate. After incubating at 25°C for 5 min in the dark, 60 μL of 7.5% (w/v) Na_2_CO_3_ was added to each well and incubated at room temperature for 30 min. The absorbance of the reaction mixture was measured at 765 nm using a microplate reader (BioTek™ Synergy™ H4 Hybrid Microplate Reader) [24, 25].

### Proteolytic digestion assay

Pepsin and proteinase K were used for proteolytic digestion. The proteolytic digestion assays were performed as previously described with minor modifications [24, 26]. Briefly, the proteins were incubated with either proteinase K (Invitrogen, Thermo Fisher Scientific™, California, USA) or porcine pepsin (Sigma–Aldrich, Missouri, USA) at an enzyme-to-protein ratio of 1:25. The mixture was incubated at 37°C for 12 h, followed by heat inactivation at 100°C for 10 min. The digested proteins were then used for antifungal activity testing and the residual proteins were detected using sodium dodecyl sulfate-polyacrylamide gel electrophoresis (SDS-PAGE).

### Sodium dodecyl sulfate-polyacrylamide gel electrophoresis

The SYP protein pattern was determined using the SDS-PAGE. Five micrograms of each SYP1–4 sample were loaded into 4%–20% NuPAGE Novex Bis-Tris 1.0 mm mini gels (Invitrogen, CA, USA). A protein molecular weight marker (Thermo Scientific™, Rockford, IL, USA) was included in each run. The electrophoresis was performed using a constant voltage (60–200V) for approximately 1–4 h. Subsequently, the fingerprint profile of the segregated SYP proteins (SYP protein pattern) was visualized using Coomassie Brilliant Blue staining (Thermo Scientific™ Imperial™ Protein).

### Antifungal activity testing

#### Fungal strains and culture conditions

Four fungal reference strains and six animal clinical fungal isolates, that were isolated and identified at the Small Animal Teaching Hospital (Akkhraratchakumari Veterinary College, Walailak University, Thailand), were chosen. These fungal strains included three yeasts, viz. (1) *Candida albicans* ATCC90028 (CA), (2) *Cryptococcus neoformans* ATCC90112 (CN), and (3) *Malassezia pachydermatis* clinical isolate (MP), and two dimorphic fungi, namely, (1) *T. marneffei* (mold phase) ATCC200051 (TMM) and *T. marneffei* (yeast phase) ATCC200051 (TMY) and (2) *Sporothrix schenckii* clinical isolate (mold phase) (SCM) and *Sporothrix schenckii* clinical isolate (yeast phase) (SCY). In addition, five molds – *Aspergillus fumigatus* NCPF7367 (AF), *Microsporum gypseum* clinical isolate (MG), *Microsporum canis* clinical isolate (MC), *Trichophyton mentagrophytes* clinical isolate (TME), and *Trichophyton rubrum* clinical isolate (TR) – were used in this study.

The yeast colonies of CA, CN, and MP were inoculated into Sabouraud dextrose broth and cultured at 37°C with continuous shaking (24 for CA and 48 h for CN and MP). The yeast-like colony of dimorphic fungi (TMY and SCY) was shaken and incubated in a Brain heart infusion broth at 37°C for 96 h. The number of yeast cells was enumerated using a hemacytometer and the culture was adjusted to 10^6^ cells/mL for yeast and 10^8^ cells/mL for yeast-dimorphic fungus. The five monomorphic filamentous fungi (*A. fumigatus*, *M. gypseum*, *T. mentagrophytes*, *M. canis*, and *T. rubrum*) and two dimorphic fungi (*T. marneffei* and *S. schenckii*) were grown on the potato dextrose agar (PDA) at 25°C for 7–14 days. The conidia were collected by scraping the colony surface, suspended in PBS-0.1% tween, enumerated, and adjusted to a final concentration of 5 × 10^5^ conidia/mL [23, 27–29].

### Agar method for antifungal activity testing

The modified agar diffusion assay was used to determine the antifungal activity of SYPs, as previously described [23, 27–29]. Briefly, Muller–Hinton agar (MHA) with 2% glucose and 0.5 μg/mL methylene blue (MHA + 2G + M) and MHA with 2% glucose agar (MHA + 2G) plates were prepared for fungal cultures. Yeast suspensions were then spread on the surface of MHA + 2G + M agar plates. Conidia suspension of each fungus was spread on MHA + 2G agar plates. Then, using a sterile borer (5 mm diameter), wells were created in the agar plates. The well was filled with a 100 μL solution containing 500 μg of each protein extract – SYP1–4. The agar plates were incubated at an appropriate growth condition for each fungus as follows: *C. albicans*, 37°C for 24 h; *C. neoformans*, 37°C for 48 h; *M. pachydermatis* 30°C for 48 h; yeast phase of *T. marneffei*, 37°C for 96 h, and mold phase of *T. marneffei*, 25°C for 72 h; mold phase of *S. schenckii* 25°C for 5 days, and yeast phase of *S. schenckii* 37°C for 96 h; *A. fumigatus*, 25°C for 72 h; and *M. gypseum*, *M. canis*, *T. mentagrophytes*, and *T. rubrum* 25°C for 5–7 days. The antifungal effects were reported as follows: −, no suppression; +, suppression, with detectable inhibition zones around the wells. All experiments were performed in duplicates. Qualitative control: growth control (media + test organism) were conducted to compare the active growth of microorganisms with that occurring in the presence of SYP1–4. Media control was done to detect the sterility of the agar plate test. Drug control or positive control (10 μg/mL amphotericin B) was conducted to compare the efficacy of the antifungal drug with that of SYP1–4. Negative controls for SYPs included testing the antifungal effect of the inactivated enzyme suspension without the hydrolysate protein fraction. Therefore, 10 mM sodium acetate buffer pH = 5.2, 10 mM sodium acetate buffer pH 3.7 with and without pepsin, and 1× PBS pH = 7.4 with and without proteinase K were used as negative controls of SYP1 and SYP2, SYP3, and SYP4, respectively.

### Determination of the minimal inhibitory concentration (MIC) and MFC

The MIC values were determined using modified M27-A4 and M38-A3 broth microdilution assay protocols [24, 30, 31]. The final protein concentration of the SYP1–4 test solutions was adjusted in the range of 0.5–256 μg/mL. 50 mL of each protein solution containing 1–512 μg/mL protein was placed into sterile 96-well microtiter plates. An equal volume of fungal suspensions in RPMI 1640 medium (5 × 10^3^ cells/mL for yeast, 5 × 10^4^ conidia/mL for mold, and 5 × 10^5^ conidia/mL for dermatophytes) was added to each well, making the final concentration of precipitated protein 0.5–256 μg/mL. Plates were incubated in the conditions appropriate for the given fungus (described in “Agar method for antifungal activity testing” section). Subsequently, resazurin (0.18% w/v, final concentration; Sigma–Aldrich) indicator was added to each well. The fungal inhibition results were interpreted based on a resazurin color change and the absorbance values at 600 nm. The MIC values were determined as the lowest concentration of the SYP1–4, which could inhibit the fungal growth. If there was no inhibition at the maximum tested concentration (256 μg/mL), the MIC was recorded as >256 μg/mL.

The minimal fungicidal concentrations (MFCs) were determined for each positive MIC well (purple-dark blue color) combination as previously described [23, 24, 32]. Briefly, the MFCs were determined by dropping 10 μL of SYP1–4, at concentrations used in the MIC wells and higher, onto the surface of Sabouraud dextrose agar (SDA) plate for yeast, and a PDA plate for mold. The plates were incubated at 37°C for 48 h. The lowest protein concentration of the SYP1–4 that showed no visible growth of fungi on the agar plates was recorded as the MFC. If colonies were observed at the maximum concentration, MFC value was recorded as >256 μg/mL.

### Hemolytic assay

A hemolytic assay was performed to test the safety of SYP4 on animal red blood cells (RBCs). The animal experiments were approved by Walailak University Institutional Animal Care and Use Committee (WU-IACUC, Walailak University, Thailand) and authorized all animal care methods (WU-ACUC-65037). Fresh canine blood from a consenting healthy donor was collected in Blood Collection Tubes (BD Vacutainer^®^, Beckton Dickinson, São Paulo, Brazil) and centrifuged at 850× *g* at 4°C for 5 min. The plasma was removed and the pellet containing the RBCs was washed 3× with a sterile 1 × PBS pH 7.4. The pellet was resuspended in a calculated volume of PBS to obtain a 2% (*v/v*) suspension of RBCs. One hundred microlitter aliquot of this suspension was transferred to sterile microtubes containing 100 μL of individual SYP4 solutions (at concentrations 16 × MIC, 8 × MIC, 4 × MIC, and 2 × MIC). The final concentrations of RBCs and SYP4 in the hemolytic assay were 1% (*v/v*) and 8 × MIC, 4 × MIC, 2 × MIC, and MIC, respectively. After incubation (37°C, 60 min), the reaction was stopped by placing on ice for 5 min. Then, the microtubes were centrifuged at 3000× g for 2 min, the supernatants were collected and transferred to 96-well flat bottom clear plates. Hemoglobin released from lysed erythrocytes was estimated at 540 nm using the microplate reader (BioTek™ Synergy™ H4 Hybrid Microplate Reader). Untreated RBCs suspensions and RBCs treated with distilled water were used as negative and positive controls, respectively [33]. All tests were performed in triplicates. The % hemolysis was determined using the formula,


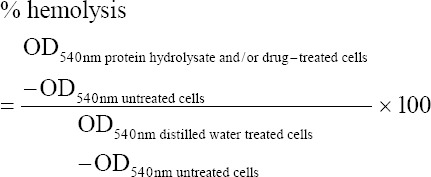


### Statistical analysis

All data were subjected to a one-way analysis of variance, followed by Duncan’s multiple range test to verify if the differences between the means were significant. The normal distribution and homogeneity of variance of the data were checked by the Shapiro–Wilk and Levene’s tests, respectively. Variability in the data was expressed as mean ± standard error of the mean; p ≤ 0.05 was considered significant. The data were analyzed using the Statistical Package for the Social Sciences version 16.0 (SPSS Inc, Chicago, USA).

## Results

### Sangyod rice seed protein and phenolic compound contents and SYP1–4 protein profiles

The total protein in the Sangyod rice crude protein supernatant SYP1 was 394.57 μg/kg of rice seed. The total phenolic content in SYP1 was 134.40 μg/kg of rice seed. The total proteins to total phenolic compounds ratio was 2.94. These phenolic compounds were found to have very low antifungal activity (MIC of 0.170 mg) when tested using 500 μg protein equivalent of SYP.

**Figure-1:**
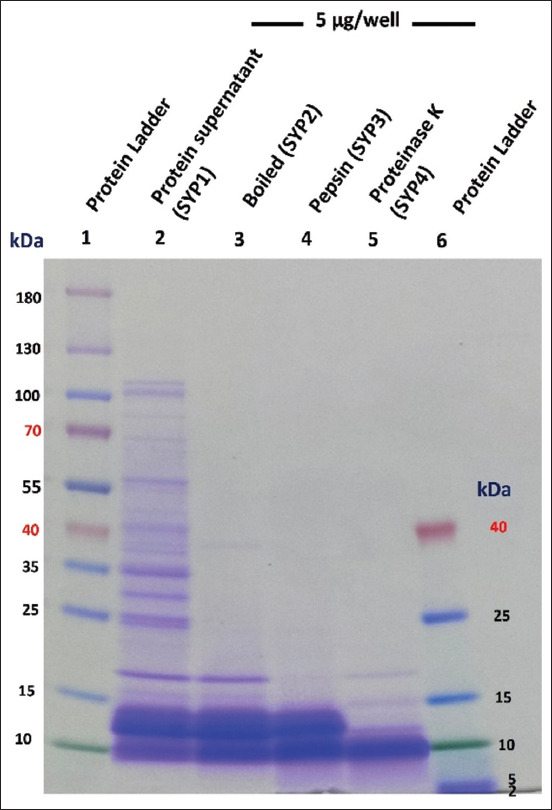
Detection of Sangyod rice seed protein patterns using sodium dodecyl sulfate polyacrylamide gel electrophoresis (SDS-PAGE). Five micrograms of each protein extract were separated in SDS-PAGE gel (4–20% gradient acrylamide gel, Invitrogen, CA, USA) and stained with Coomassie blue (Thermo Scientific™ Imperial™ Protein, Rockford, IL, USA). Lane 1: Standard protein marker *(*Thermo Scientific, Rockford, IL, USA). Lanes 2: Sangyod rice seed protein extract (SYP1), Lane 3: Heat-treated Sangyod rice seed protein, Lane 4: Pepsin digestion of SYP1, Lane 5: Proteinase K digestion of SYP1, Lane 6: Low range protein marker *(*Thermo Scientific, Rockford, IL, USA).

On the SDS-PAGE gel, the SYP1 proteins were segregated in the size range of 10–140 kDa. Three dominant bands were detected at 15–25 kDa, 10–15 kDa, and 10 kDa. The heat-treated proteins in SYP2 (SYP1 boiled at 100*°*C for 10 min) separated into only these three dominant bands, indicating that heating destroyed most of the proteins in SYP. These three bands, therefore, were heat-resistant. The SYP1 was further digested with pepsin and proteinase K to give SYP3 and SYP4, respectively. The SYP3 proteins, which were pepsin digested, separated, differently, below 15 kDa, showing a distinct hydrolysate protein pattern. For the proteinase K-digested SYP4, only a major peptide band at approximately 10 kDa was observed (Figure-1). The protein at 10 kDa mark (Figure-1) is probably the major protein involved in the antifungal activity.

### Protein hydrolysates SYP2–4 showed antifungal activity whereas the crude protein extract SYP1 showed none

**Figure-2:**
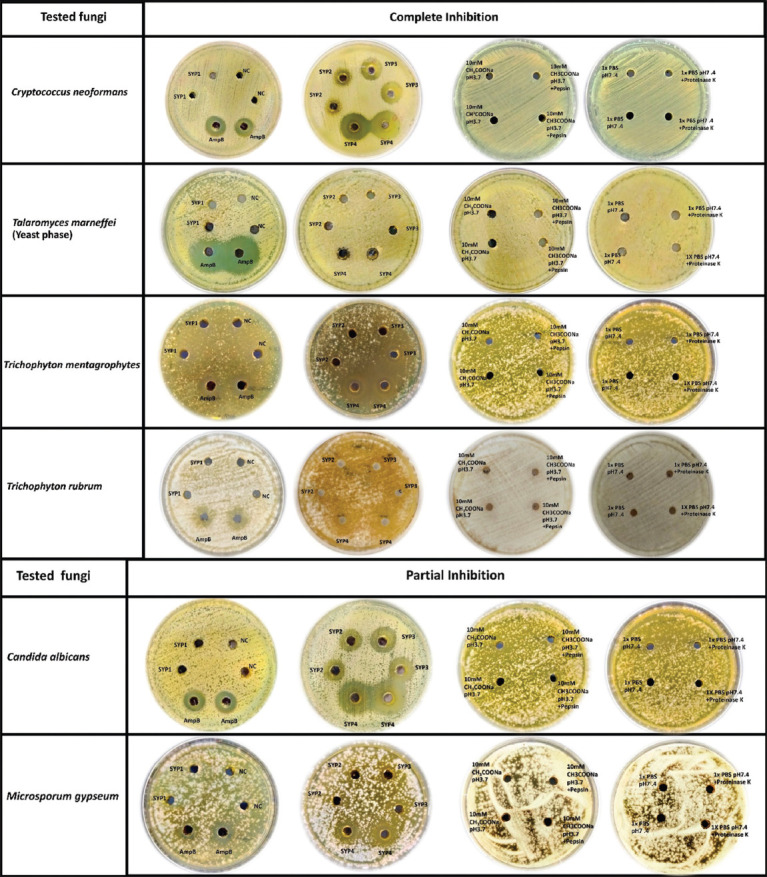
Antifungal activity of a protein extract (SYP1), heat-treated protein (SYP2), pepsin-hydrolyzed protein (SYP3), and proteinase K-hydrolyzed protein (SYP4) from Phatthalung Sangyod rice (*O. sativa* L.) seed. They were evaluated by agar well diffusion method against ten pathogenic fungi. The fungal cells were spread onto the surface of the agar medium, and the tested reagent was used to fill the appropriate wells. Positive (10 μg/mL Amphotericin B; AmpB) and negative (NC; 10 mM CH_3_COONa pH = 5.2) controls are shown. Meanwhile, 10 mM CH_3_COONa pH = 3.7 and 1 × PBS pH = 7.4 with and without enzyme were negative controls of SYP3 and SYP4, respectively. Negative controls did not affect fungal growth. Plates were incubated at 25–37°C for 3–7 days. NC: Negative control.

To confirm antifungal activity and its molecular source, the agar diffusion method tested SYP crude protein extract (SYP1) and its protein hydrolysates (SYP2–4) against ten medically important fungi. The inhibition zones representing antifungal activity (Figure-2) were recorded in millimeters, as shown in Table-1. Here, three types of inhibition zones were identified. First, the “complete inhibition” zone consists of a clear fungal-free area (adjacent to the test well) with a dense fungal growth area around it. Second, the semiclear “partial inhibition” zone, having some colonies of either resistant fungi or regrown fungi (after the decomposition of the test agent) grows within this zone. Third, the opaque “no inhibition” zone with a lawn of fungi adjacent to the test well, lacks clear or incomplete zones.

**Table-1:** Antifungal activity of protein extract and its protein hydrolysates from Phatthalung Sangyod rice seed against ten pathogenic fungi.

Sangyod rice seed protein	Antifungal activity (mean ± SD of inhibition zone diameter (mm))											

	CA	CN	AF	TMM	TM	MG	MC	TME	TR	SCM	SCY	MP
SYP1	-	-	-	-	-	-	-	-	-	-	-	-
SYP2	PI (13.50 ±0.58)	+ (8.50 ± 0.58)	-	-	-	-	-	-	-	-	-	-
SYP3	PI (16.00 ± 0.82)	+ (15.50±0.58)	-	-	-	-	-	-	-	-	-	-
SYP4	PI (21.75 ± 0.50)	+ (20.25 ± 0.50)	-	-	+ (8.00 ± 0.58)	PI (14.25 ± 0.96)	-	+ (14.25 ± 0.50)	+ (11.00 ± 0.00)	-	-	-
Negative control: 10 mM CH_3_COONa pH = 5.2	-	-	-	-	-	-	-	-	-	-	-	-
10 mM CH_3_COONa pH = 3.7	-	-	-	-	-	-	-	-	-	-	-	-
10 mM CH_3_COONa pH = 3.7+Pepsin	-	-	-	-	-	-	-	-	-	-	-	-
1 × PBS pH 7.4	-	-	-	-	-	-	-	-	-	-	-	-
1 × PBS pH 7.4 + Proteinase K	-	-	-	-	-	-	-	-	-	-	-	-
Positive control: Amphotericin B (10 µg/mL)	+ (15.00 ± 0.50)	+ (15.00 ± 0.50)	+ (10.25 ± 0.50)	+ (20.25 ± 0.50)	+ (18.00 ± 1.15)	+ (14.00 ± 0.00)	+ (19.25 ± 0.50)	+ (13.50 ± 0.58)	+ (14.25 ± 0.50)	+ (19.00 ± 0.00)	+ (15.00 ± 0.00)	+ (17.00 ± 0.00)

+=Complete inhibition, -=No inhibition, PI=Partial inhibition, CA=*Candida albicans* ATCC90028, CN=*Cryptococcus neoformans* ATCC90112, AF=*Aspergillus fumigatus* NCPF7367: TMY=T*alaromyces marneffei* (yeast phase) ATCC200051, TMM=*Talaromyces marneffei* (mold phase) ATCC200051, MG=*Microsporum gypseum* Clinical isolate, MC=*Microsporum canis* Clinical isolate, TME=*Trichophyton mentagrophytes* Clinical isolate, TR=*Trichophyton rubrum* Clinical isolate, SCM=*Sporothrix schenckii* Clinical isolate (mold phase), SCY=*Sporothrix schenckii* Clinical isolate (yeast phase), MP=*Malassezia pachydermatis* Clinical isolate. The value represents in means±SD from three independent experiments

Specifically, the heat-treated protein (SYP2) and protein hydrolysates (SYP3 and SYP4) from Sangyod rice seed could suppress the growth of at least one tested fungus, but the crude protein (SYP1) did not. SYP2–4, especially SYP4, showed strong antifungal activity against *C. neoformans*, yeast-*T. marneffei*, *T. mentagrophytes*, *T. rubrum*, and partial inhibition on *C. albicans* and *M. gypseum*.

### Determination of the minimal inhibitory and fungicidal concentration (MIC/MFC)

**Figure-3:**
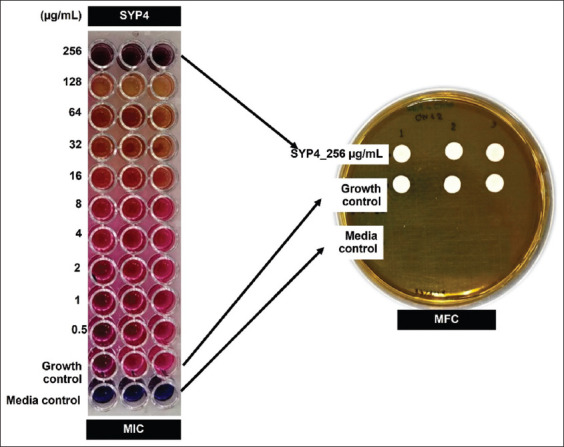
Minimal inhibitory concentration (MIC) and minimal fungicidal concentration (MFC) values of the proteinase K-hydrolyzed protein (SYP4) from Sangyod rice seed on the anti-*Cryptococcus neoformans* activity. *Cryptococcus neoformans* yeast cells (5 × 10^3^ cells/mL) were treated with SYP4 in the dilution range of 0.5–256 μg/mL and incubated for 48 h at 37°C. The pink and purple-dark blue colors in MIC wells indicated inhibition and no inhibition of *C. neoformans* growth, respectively. It indicated the MIC value. The MFC value was indicated by the absence of *C. neoformans* colony growth on sabouraud dextrose agar (SDA) plates. The control growth of *C. neoformans* was shown on the white spots on the SDA surface.

The MIC and MFC of SYP2, SYP3, and SYP4 needed to inhibit *C. neoformans* were estimated. In addition, MIC and MCF of SYP4, needed to inhibit *T. rubrum*, *T. mentagrophytes*, and *T. marneffei* (yeast phase) were also estimated. Based on the positive agar diffusion plate assay results, we used a final SYP2–4 proteins concentration of 500 μg for evaluating MIC and MFC. Against *C. neoformans* SYP4 had the MIC/MFC values of 256/>256 µg/mL, while SYP2 and SYP3 were >256/>256 µg/mL (Table-2). Furthermore, MIC of SYP4 against *T. metagophytes*, *T. rubrum*, and *T. marneffei* was undetectable in the tested concentration range of 0.5–256 μg/mL (Table-2 and Figure-3).

**Table-2:** Minimal inhibitory concentration and MFC values of the Sangyod rice seed protein and protein hydrolysates on pathogenic fungi (µg/mL).

Sangyod rice seed protein hydrolysates	Tested fungi (MIC/MFC)			

	*C. neoformans*	*T. mentagrophytes*	*T. rubrum*	*T. marneffe*i (yeast phase)
SYP2	>256/>256	NT/NT	NT/NT	NT/NT
SYP3	>256/>256	NT/NT	NT/NT	NT/NT
SYP4	256/>256	>256/NT	>256/NT	>256/NT
Positive control: Amphotericin B (0.03125–16 µg/mL)	0.5/2	0.5/8	0.5/8	0.25/1

NT=Not tested, MIC=Minimal inhibitory concentration, MFC=Minimal fungicidal concentration, *C. neoformans=Cryptococcus neoformans, T. mentagrophytes=Trichophyton mentagrophytes,* *T. rubrum=Trichophyton rubrum, T. marneffei=Talaromyces marneffei*

### SYP4 is non-toxic to canine RBCs

**Figure-4:**
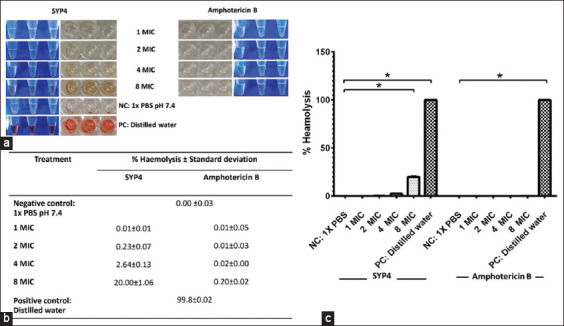
*In vitro* canine hemolytic activity of proteinase K-hydrolyzed protein from Sangyod rice seed. Red blood cells (RBCs) were obtained from a healthy dog. The diluted canine RBCs were incubated with SYP4 at 37°C for 1 h. (a) Photographs of each SYP4 minimal inhibitory concentration on canine erythrocyte cell lysis (centrifugation, 3000× *g*, 2 min to a completely clear solution); red color in tube supernatants indicates damaged RBCs (release of hemoglobin); and the supernatants (100 μL) were transferred to 96-well flat bottom clear plates. (b) The supernatant was spectrophotometrically analyzed, the absorbance spectra were obtained, and the results were evaluated at 540 nm. (c) The percentage of hemolysis of RBCs in the untreated control was compared with positive control and various concentrations of SYP4. Distilled water and phosphate-buffered saline were used as positive and negative controls, respectively. *p *≤* 0.05 (n = 3 replicates).

Cytotoxicity of the protein hydrolysate SYP4 on canine RBCs was tested. The canine RBCs were incubated with various concentrations of SYP4 at 37°C for 1 h and the extent of hemolysis was determined. Notably, none of the tested concentrations of SYP4 caused significant hemolysis; <20% hemolysis was seen. Specifically, the hemolysis was (in %) 20.00 ± 1.06, 2.64 ± 0.13, 0.23 ± 0.07, and 0.01 ± 0.01 at SYP4 concentrations of 8 × MIC (2,048 μg/mL), 4 × MIC (1,024 μg/mL), 2 × MIC (512 μg/mL), and MIC (256 μg/mL), respectively (Figure-4).

## Discussion

Sangyod “dark-red pigmented” rice is an indigenous geographical indication tagged product of Thailand. It is rich in nutrients, such as proteins, carbohydrates, fibers, vitamins (B1, B2, and B6), iron, calcium, and phosphorous [3–7]. It is also rich in natural antioxidants. Due to high content of the major antioxidants, such as phenolic compounds and anthocyanin, most Sangyod rice studies have focused on its antioxidant activity. Its antibacterial activity is also known [34]. Only one study by Suesattayawong *et al*. [22] reports that Sangyod rice seed has antifungal activity against the *S. oryzae*, a plant pathogen. However, the antifungal activity of Sangyod rice seed extracts – on medically important fungi causing zoonoses – has not yet been studied.

Sangyod rice seed crude protein extract (SYP1) did not show antifungal activity against any of the ten tested pathogenic fungal strains. SYP1 and the protease enzymes (negative control) lacked antifungal activities, indicating that neither the crude Sangyod protein extract nor the residual Sangyod phenolic compounds possess antifungal properties. Surprisingly, the heated hydrolysate SYP2 demonstrated antifungal activity. Thus, the cooked Sangyod rice seed may continue to contain thermophilic or heat-tolerant proteins with antifungal properties. SYP2 inhibited the growth of some strains of fungi, especially encapsulated fungal pathogens, *C. neoformans* and partially inhibited *C. albicans*, indicating that Sangyod rice can promote health by combating fungal infections. High-temperature potently turns inactive protein precursors into bioactive proteins with antifungal activity. Alternatively, the heat-tolerant proteins in the Sangyod rice seed extract turn into bioactive peptides on heating [35].

Pepsin-hydrolysate SYP3 also possessed antifungal properties. It exhibits similar, but stronger antifungal activity when compared to SYP2, possibly due to pepsin-mediated lysis of precursor proteins into bioactive peptides with antifungal activity. Moreover, SYP2 and SYP3 actions are specific to yeast strains (*C. neoformans* and *C. albicans*). Even though yeast and filamentous fungi belong to the same family, their main cell wall composition is different [36]. Further, the major fungal cell wall components are not present in humans, making these fungi an excellent target for testing clinical antifungals and designing immunotherapies [36, 37]. Garcia-Rubio *et al*. [36] and Struyfs *et al*. [37] proposed that ideal antifungals which interfere/disrupt fungal cell walls are highly fungi-specific. Agreeingly, many effective antimicrobials, such as AFPs, kill microbes by rapid physical disruption of microbial membranes. Moreover, numerous AFPs interact with the surface of fungal cells and affect intracellular targets [37]. This mode of inhibition must be confirmed through additional research. Interestingly, we found that proteinase K-treated hydrolysate (SYP4) inhibited fungi more effectively than heat-treated (SYP2) and pepsin-treated (SYP3). SYP4 inhibited more diverse species of fungi causing mycoses, such as cutaneous pathogenic fungi (*T. mentagrophytes* and *T. rubrum*), opportunistic fungi (*C. neoformans*), and dimorphic fungus (*T. marneffei*). Therefore, SYP4 could contain a potential antifungal peptide with diverse applications.

We found that the heat- and enzymatic hydrolysis methods are very effective in synthesizing bioactive compounds from Sangyod rice seeds. Du *et al*. [18] concluded that protease activity can release bioactive peptides such as an antifatigue peptide, antihypertensive peptide, antioxidant peptide, and antibacterial peptide – from wheat germ. Here, the Sangyod rice peptides derived from enzymatic (pepsin or proteinase K) hydrolysis have more powerful antifungal activity than those obtained by heating. Moreover, pepsin is FDA-approved for use in foods at levels within the stipulated current good manufacturing practices guidelines [38]. Proteinase K can potentially generate bioactive peptides from various plants [18, 19]. Here, the bioactive peptides generated using alkaline conditions (proteinase K, SYP4) have a better antifungal effect than those generated using acidic conditions (pepsin, SYP3). Consistently, Karami *et al*. [19] reported that the type of peptidase (proteinase K, pepsin, or alcalase) used, influences the antioxidant, antiproliferative, ACE-inhibiting, antiproliferative, and likely additional biological activities of the plant hydrolysates. Hence, proteinase K-treated SYP4 hydrolysate has more potent antifungal activity here than pepsin-treated SYP3. Probably, because proteinase K splits proteins at their hydrophobic, aromatic, and aliphatic amino acid residues [39]. In addition, proteinase K digests proteins into smaller fragments of <10 kDa. Hence, proteinase K can be regarded as a potential peptidase for the hydrolysis of Sangyod rice seed and wheat germ in generating bioactive peptides [19].

SYP4 has not been functionally characterized here. Future research must focus on the functional and molecular characterization of SYP4 to discern its free amino acids and polypeptides composition. SYP4 should be further purified to identify and test its individual components for their antifungal and other bioactive properties. Further, verifying if the bioactivities of SYP2, SYP3, and SYP4 directly affect the fungi cell wall, would clarify if they are ideal antifungals. Taken together, we demonstrate here, that the molecules imparting antifungal properties to Sangyod rice protein extracts are either peptides or free amino acids. Importantly, we find that SYP4 is not toxic to canine RBCs, indicating that it is safe for use in clinical applications. In addition, methods to prepare standardized antifungal formulas using cooked or raw rice must be researched.

## Conclusion

This is the first study to report the antifungal activities of the heat-treated protein hydrolysates of the Sangyod rice seed. Sangyod protein hydrolysates contain non-toxic potential antifungals. The putative Sangyod antifungals inhibit various human and animal pathogenic fungi. Alkaline protease K-treated Sangyod rice protein hydrolysate (SYP4) had the most antifungal bioactivity. Daily consumption of Sangyod Phatthalung rice might improve animal and human health and help fight fungal infections.

## Authors’ Contributions

JJ, SR, and MP: Conceptualization. JJ, PR, KP, WM, CB, IT, and NT: Methodology and investigation. JJ and MP: Writing-original draft preparation and writing-review and editing. JJ: Funding acquisition. All authors have read, reviewed, and approved the final manuscript.
